# Ecological Modelling of Potential Habitats for Indian Mackerel (*Rastrelliger* spp.) in the Western of Banda Sea using an Artificial Neural Network Approach

**DOI:** 10.21315/tlsr2025.36.3.9

**Published:** 2025-10-31

**Authors:** Alfira Yuniar, Mukti Zainuddin, Safruddin, Muzzneena Ahmad Mustapha, Rachmat Hidayat, Siti Khadijah Srioktoviana

**Affiliations:** 1 Fisheries Department, Faculty of Marine Science and Fisheries, Hasanuddin University, Jl. Perintis Kemerdekaan KM 10 South Sulawesi, 90245, Makassar, Indonesia; 2 Faculty of Science and Technology, Universiti Kebangsaan Malaysia, 43600 UKM Bangi, Selangor, Malaysia

**Keywords:** Indian Mackerel, ANN Algorithm, Spatial Modelling, Remote Sensing, Oceanographic Condition, Ikan Kembung, Algoritma ANN, Pemodelan Spasial, Penderiaan Jauh, Kondisi Oseanografi

## Abstract

Indian mackerel (*Rastrelliger* spp.) is a species with high catch volumes, amounting to approximately 451.750 tonnes over five years. This substantial yield holds significant potential for local communities, making sustainable utilisation crucial. This study focuses on the fishing season of Indian mackerel (*Rastrelliger* spp.) and the development of a habitat suitability model in the waters of the western of Banda Sea, Indonesia. The Fishing Season Index (FSI) method identified November as the peak fishing season, with the highest CPUE recorded at 220 kg trip^−1^. During this period, stable salinity levels were observed, which supported the reproductive processes of Indian mackerel. Additionally, high rainfall and strong winds facilitated local upwelling, influencing currents and bringing nutrients to the surface, which were consumed by mackerel larvae. The ANN (Artificial Neural Network) models used to estimate potential fishing zones for Indian mackerel demonstrated high accuracy, with an error rate of just 1.12%. The analysis revealed that salinity and currents were the most influential environmental parameters, contributing 16% and 14% to catch success during the peak fishing season with salinity levels at 34.2 psu and current velocity at 3.29 cm s^−1^. The implementation of this model in analysing Indian mackerel habitats and their relationship with environmental factors supports data and technology-driven fisheries management. This study also introduces a novel integration of the Fishing Season Index (FSI) method and ANN modelling to simultaneously identify peak fishing seasons and predict potential fishing zones based on dynamic oceanographic parameters. The application of machine learning in this model enables the identification of non-linear relationships between environmental variables and fish distribution with high accuracy, representing a significant advancement in predictive habitat modelling for Indian mackerel in Indonesian waters. This approach contributes to sustainable fisheries resource management and aligns with the achievement of SDG 14 in Indonesia.

## Abstract (fr)

**Abstrak:**

Ikan kembung (Rastrelliger spp.) merupakan spesies dengan jumlah tangkapan yang tinggi iaitu sekitar 451.750 tan dalam tempoh lima tahun. Jumlah ini berpotensi besar untuk masyarakat setempat, menjadikan penggunaan yang mampan sangat penting. Kajian ini menumpukan kepada musim penangkapan ikan kembung (Rastrelliger spp.) dan pembangunan model kesesuaian habitat di perairan barat Laut Banda, Indonesia. Kaedah Indeks Musim Penangkapan (Fishing Season Index/FSI) mengenal pasti bulan November sebagai musim puncak penangkapan dengan nilai CPUE tertinggi sebanyak 220 kg trip^−1^. Pada tempoh tersebut, paras saliniti yang stabil telah diperhatikan, menyokong proses pembiakan ikan kembung. Selain itu, hujan lebat dan angin kencang membolehkan berlakunya fenomena “upwelling” tempatan yang mempengaruhi arus dan membawa nutrien ke permukaan laut untuk dimakan oleh larva ikan kembung. Model ANN (Artificial Neural Network) yang digunakan untuk menganggar zon penangkapan ikan kembung menunjukkan ketepatan yang tinggi dengan kadar ralat hanya 1.12%. Analisis mendapati saliniti dan arus merupakan parameter persekitaran yang paling mempengaruhi kejayaan tangkapan menyumbang masing-masing 16% dan 14% semasa musim puncak dengan paras saliniti pada 34.2 psu dan kelajuan arus pada 3.29 cm s^−1^. Pelaksanaan model ini dalam menganalisis habitat ikan kembung dan hubungannya dengan faktor persekitaran menyokong pengurusan perikanan berasaskan data dan teknologi. Kajian ini juga memperkenalkan integrasi baru kaedah Indeks Musim Memancing (FSI) dan pemodelan ANN untuk mengenal pasti musim penangkapan kemuncak secara serentak dan meramalkan zon penangkapan ikan yang berpotensi berdasarkan parameter oseanografi dinamik. Aplikasi pembelajaran mesin dalam model ini membolehkan pengenalpastian hubungan bukan linear antara pembolehubah persekitaran dan taburan ikan dengan ketepatan yang tinggi, mewakili kemajuan yang ketara dalam pemodelan habitat ramalan untuk ikan kembung di perairan Indonesia. Pendekatan ini menyumbang kepada pengurusan sumber perikanan yang mampan dan sejajar dengan pencapaian SDG 14 di Indonesia.

**HIGHLIGHTS**

- The study identified November as the peak fishing season for Indian mackerel, with the highest catch per unit effort (220 kg per trip). This peak season is supported by stable salinity, heavy rainfall and upwelling currents that enhance nutrient availability and reproduction.
- The results showed that salinity and ocean currents are the most influential oceanographic factors, contributing 16% and 14%, respectively, to catch productivity. These conditions play a vital role in determining the distribution and abundance of Indian mackerel.
- The application of an Artificial Neural Network (ANN) model demonstrated high accuracy with only a 1.12% error rate in predicting potential fishing zones. By integrating the Fishing Season Index (FSI) and ANN.

## INTRODUCTION

Capture fisheries play a pivotal role in bolstering food security and the economy of coastal communities in Indonesia (Fuadi & Wiryawan 2018). Among the fish species with significant economic value, mackerel (*Rastrelliger* spp.) stands out as a primary commodity in the small pelagic fisheries sector (Nurdin *et al*. 2015; Kamaruzzaman *et al*. 2021). The high demand for mackerel highlights the urgency of implementing sustainable fisheries management, particularly due to evidence of stock fluctuations and localised population declines in several regions (Shaari & Mustapha 2018; Lakhnigue *et al*. 2019; Srioktoviana *et al*. 2024). Recent studies have shown that mackerel populations, including *Rastrelliger* spp., are increasingly vulnerable to overfishing and environmental variability, which may threaten long-term availability if not properly managed (Putri & Zainuddin 2019a; Huang *et al*. 2022; Nair *et al*. 2023). Therefore, integrating scientific approaches to enhance fishing efficiency while protecting critical habitats is essential to ensure the sustainability of mackerel fisheries.

The identification of an appropriate fishing season is a critical determinant in the success of fishing operations (Ben-Hasan *et al*. 2019; Putri & Zainuddin 2019b). Traditional methods of determining fishing seasons (Kurniawati *et al*. 2015) often rely on local knowledge and the experiental wisdom of fishers, which may prove inadequate in addressing the complexities and variability of fish stocks caused by oceanographic phenomena such as the El Nino Southern Oscillation (ENSO) and global climate change (Zainuddin *et al*. 2020; Mukherjee *et al*. 2023). Consequently, technology-driven and data-centric approaches are increasingly relevant to support more precise and informed decision-making (Nuno *et al*. 2005; Wang *et al*. 2016). Artificial Neural Networks (ANNs) have become increasingly prominent in ecological and fisheries modelling due to their ability to capture complex, non-linear relationships among multiple environmental variables (Guisan & Zimmermann 2000; Pradana 2023; Salem *et al*. 2023). Unlike traditional statistical methods such as linear or multiple regression, which assume linearity and independence among predictors, ANNs can handle large, heterogeneous datasets and identify intricate patterns in variables such as sea surface temperature (SST), chlorophyll-a concentration, salinity, and ocean currents (Zainuddin 2011; Yuniar *et al*. 2024; Wang *et al*. 2025). These advantages make ANNs particularly well-suited for modelling dynamic and spatially heterogeneous marine ecosystems, where fish behaviour is strongly influenced by the interplay of various environmental factors.

In addition, ANN-based models have demonstrated superior predictive accuracy and resilience under uncertain and variable conditions when compared to conventional approaches (Zhang & Zimba 2017; Prasad *et al*. 2023). Their application in fisheries not only enhances the precision of fish distribution and fishing season forecasts but also contributes to sustainability by informing more adaptive harvesting strategies and reducing the risk of overexploitation (Torri *et al*. 2018; Ibrahim *et al*. 2023). This study aims to develop a predictive model for the fishing season of Indian mackerel (*Rastrelliger* spp.) in the western part of the Banda Sea using an ANN algorithm. By incorporating dynamic oceanographic parameters into the modelling framework, this research seeks to contribute to data-driven fisheries management and support the implementation of sustainable fishing policies aligned with SDG 14 in Indonesia. Therefore, this study represents a strategic effort to enhance both the productivity and sustainability of the capture fisheries sector in the region.

## MATERIALS AND METHODS

### Study Area

**FIGURE 1:**
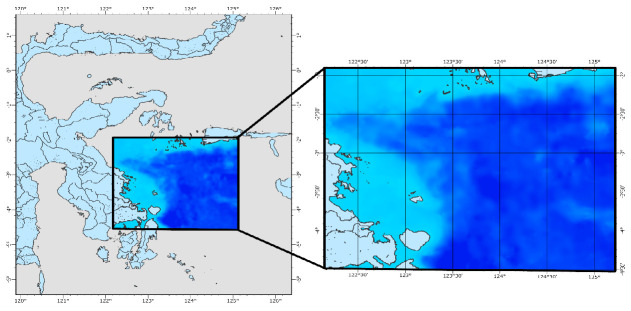
The research location is in the western of Banda Sea which is included in the Coral Triangle area, Indonesia.

This study was conducted in the western part of the Banda Sea, located within the geographical coordinates of 120°E to 126°E and 2°S to 5°S (Fig. 1). This area is an integral part of Indonesia’s marine ecosystem and is globally recognised as one of the regions with the highest marine biodiversity (Aryanti *et al*. 2018; Ministry of Environment and Forestry 2021). The Banda Sea lies within the Coral Triangle, which is widely known as the epicentre of global marine biodiversity (Veron *et al*.2009). The western Banda Sea also serves as a critical fishing ground for small pelagic species, particularly Indian mackerel (*Rastrelliger* spp.), which have historically contributed significantly to both local and national fisheries economies (Zainuddin *et al*. 2013; Kementerian Kelautan dan Perikanan [KKP] 2022). According to data from the Ministry of Marine Affairs and Fisheries, this region consistenstly records high catch volumes of Indian mackerel, making it a strategic site for studying fishing season patterns and habitat suitability (Nurdin *et al*. 2017; Harahap *et al*. 2020). The area’s dynamic oceanographic conditions—characterised by seasonal upwelling, current variability and nutrient availability—further highlight its ecological relevance for analysing the spatial and temporal distribution of mackerel.

### Fisheries Data

Monthly data on Indian mackerel fishing and production were collected between 2019–2022. The total production of mackerel catches amounted to 451.750 tonnes with approximately 16.833 fishing trips conducted using purse seine gear. The catch results were standardised using Catch Per Unit Effort (CPUE), calculated with the following mathematical formula:


$$\text{CPUE}=\frac{\text{Catch}}{\text{Effort}}$$

Where, Catch = Indian mackerel catch (Kg) and Effort = Number of fishing attempts (Trips).

The application of CPUE in this study is critical not only as a measure of fishing efficiency, but also as an indirect indicator of fish stock dynamics in the western Banda Sea. A high CPUE value reflects effective fishing performance and suggests a sufficient stock availability, while a declining CPUE may signal early signs of overfishing pressure or changes in environmental conditions (Andrade & Garcia 1999; Liu *et al*. 2020; Canales *et al*. 2024). In addition, CPUE was used to identify the most productive fishing season by analysing monthly trends. This temporal analysis allows researchers to determine peak mackerel fishing periods and better understand their spatial distribution. Furthermore, CPUE served as a target variable in the development of the ANN model, enabling the integration of catch data with environmental parameters such as sea surface temperature, salinity and ocean currents.

### Oceanographic Parameter Data

This study utilised oceanographic parameters (SST, CHL-a, SSS, Current) obtained through in situ methods or direct field observations, as well as *ex situ* methods in the form of satellite imagery from various data providers (Table 1).

Analysing the relationship between oceanographic parameters is a key factor in understanding fish catch dynamics, particularly for small pelagic species such as Indian mackerel (*Rastrelliger* spp.) (Hidayat *et al*. 2021; Srioktoviana *et al*. 2024). Previous studies have demonstrated significant correlations between marine environmental parameters and mackerel landing patterns. For instance, seasonal variations in mackerel catches have been shown to correlate positively with chlorophyll-a concentration (CHL-a) and negatively with sea surface temperature (SST), with a one-month lag (Sekadende *et al*. 2020). In addittion, to chlorophyll-a and SST, other oceanographic parameters such as sea surfacce salinity (SSS) and ocean current also play essential roles in influencing the distribution and abundance of Indian mackerel (Yang *et al*. 2024). Variability in SSS can affect water stratification and nutrient availability, indirectly influencing primary productivity and subsequently fish distribution (Lima Bomfim *et al*. 2023). Moreover, ocean currents are key in larval transport and migration patterns, shaping spatial dynamics of pelagic fish species (Safruddin *et al*. 2014; 2018). Ignoring these factors may overlook important ecological drivers of fish aggregation and movement. Therefore, incorporating SSS and current data provides a more comprehensive understanding of the biophysical environment that governs mackerel dynamics.

To ensure the accuracy and consistency of the satellite imagery used in this study, a statistical validation process was conducted by comparing satellite-derived data with in situ measurements. The comparison employed correlation analysis, Root Mean Square Error (RMSE) and Mean Square Error (MSE) methods. The results confirmed that the satellite data used in this study were sufficiently accurate and representative to support the spatio-temporal analysis of Indian mackerel distribution in the western Banda Sea.

### Data Analysis

The analysis of seasonal data in this study employs the FSI (Fishing Season Index) calculation to determine the fishing season based on the CPUE, using the following calculations:

The subsequent FSI analysis is utilised to ascertain the Fishing Season Index (FSI) (Dajan 1986) for Indian mackerel in western of Banda Sea:

Series of CPUE within 5 years period:


$${CPUE}_{i}=n_{i}$$

Moving Average CPUE (MA):


$${MA}_{i}=\frac{1}{12}\left(\sum_{i=i-6}^{i+5}{CPUE}_{i}\right)$$

Centred CPUE moving average (CMA):


$${CMA}_{i}=\frac{1}{2}\left(\sum_{i=i-6}^{i=1}{CPUE}_{i}\right)$$

Formula AM for Monthly Average ratio:


$${AM}_{i}=\frac{{CPUE}_{i}}{{CMA}_{i}}$$

Average ratio for monthly *i* (AAM):


$${AAM}_{i}=\frac{1}{n}\left(\sum_{j=1}^{n}{AM}_{ij}\right)$$

The total monthly ratio (TAM):


$$\begin{array}{l} TAM=\sum_{i=1}^{12}{AAM}_{i}\\ CF=\frac{1200}{TAM} \end{array}$$

Fishing Season Index:


$${FSI}_{i}={AAM}_{i}\times CF$$

Where, *n_i_* = CPUE of the *i*-th order; *AM_i_* = 12-month moving average of the *i*-th order; *CMA_i_* = centred moving average of CPUE in month *i*; *AM_i_* = monthly average ratio in month *i*; *AAM_i_* = average for *i*-th month (*i* = 1, 2, 3,.., 12 and *j* = 1, 2, 3,..., *n*); TAM = total of monthly average ratios; CF = Correction factor and *FSI_i_* = Fishing Season Index in month i.

In addition, this study employed statistical forecasting analysis to generalise monthly predictions of variable X, which was derived from catch point data. To improve prediction accuracy, an ANN model with a backpropagation learning algorithm was utilised. ANN is one of the most widely used artificial intelligence-based models due to its ability to model complex nonlinear relationships with high accuracy (Wu *et al*. 2020; Kenny *et al*. 2024). The ANN model architecture in this study consisted of three layers: one input layer, one hidden layer and one output layer. The input layer received predictor variables extracted from oceanographic and catch data. The hidden layer comprised 10 neurons, determined through a trial-and-error process to achieve optimal model performance. The sigmoid activation function was used in the hidden layer, while a linear activation function was applied in the output layer, which is appropriate for continuous variable prediction tasks. The dataset was divided into two parts: 70% for training data and 30% for testing data, using a random sampling method. This partitioning aimed to objectively evaluate the model’s performance on unseen data. To evaluate the performance of the ANN model, two statistical error metrics were used: MSE and RMSE.

MSE is a commonly used metric to measure the average of the squared differences between the predicted values and the actual values. It is calculated as:


$$MSE=\frac{1}{n}\sum_{i=1}^{n}{\left(Y_{i}-{\overset{´}{Y}}_{i}\right)}^{2}$$

Where *Y_i_* = actual value and *n* = number of observation/rows.

A lower MSE value indicates that the model’s predictions are closer to the actual data. However, since MSE produces values in squared units, RMSE is also used to bring the error back to the same unit as the original data. RMSE is the square root of MSE, formulated as:


$$RMSE=\sqrt{\sum \frac{{\left(y_{i}-y_{p}\right)}^{2}}{n}}$$

Where *y_i_* = actual value, *y_p_* = predicted value and *n* = number of observation/rows.

RMSE provides a more interpretable scale for the error, making it easier to assess the predictive performance of the model in real-world units. Both metrics are sensitive to large errors, which makes them suitable for identifying models with poor generalisation. These error metrics are widely adopted in machine learning and time-series forecasting studies to ensure the reliability and robustness of predictive models (Datta & Faroughi 2023; Nicolas *et al*. 2023).

## RESULTS

### Fishing Season

**FIGURE 2:**
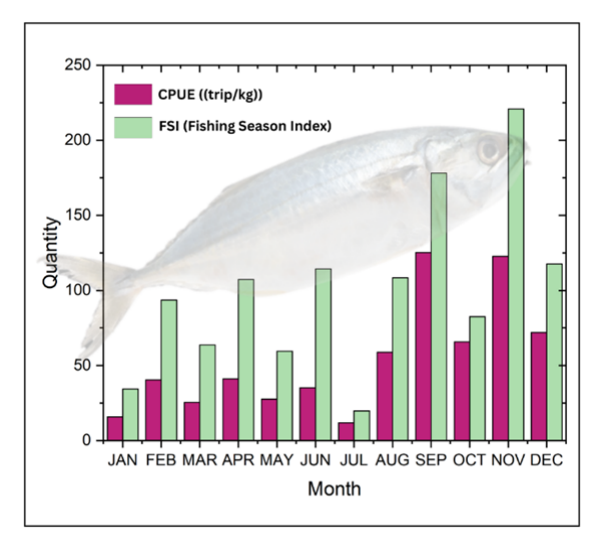
The study’s experimental setup in a greenhouse.

Based on the analysis and processing of mackerel production data from 2019–2023 (see Fig. 2), it was determined that the mackerel fishing season (*Rastrelliger* spp.) occurs in November.

In Fig. 2, the highest CPUE and FSI values are November which has the highest number of catches and intensity. Other details can be seen in Table 2.

The low fishing season occurs from January to May, characterised by low CPUE and FSI values, indicating that this period is less productive for fishing. Conversely, higher CPUE values in certain months suggest that fish populations are more abundant or easier to catch during those periods, while FSI values reflect that fishermen are actively capitalising on this time as the primary fishing season. As for August, September, November and December, these months represent the harvesting period.

**FIGURE 3:**
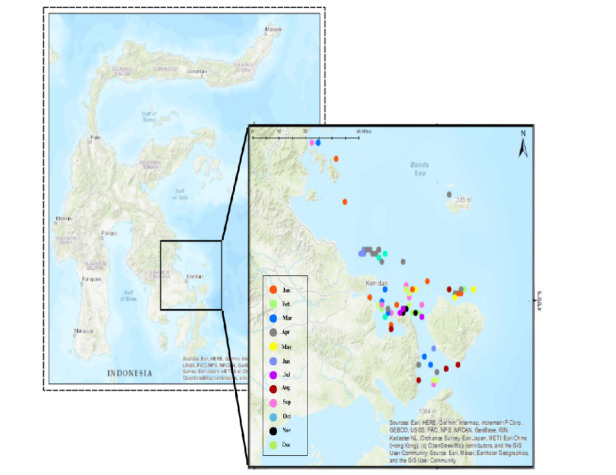
Coordinate points for mackerel fishing (*Rastrelliger* spp.) from 2019 to 2023 (five years) in the Northwest of Banda Sea.

Mackerel fishing efforts in the research area over the past 3 to 4 years have shown a minimal level of activity (Fig. 3). This indicates that the fishing efforts are relatively limited, with indications that fishermen are concentrating their efforts at the same location during this period.

### Relationship between Oceanographic Parameters and CPUE

**FIGURE 4:**
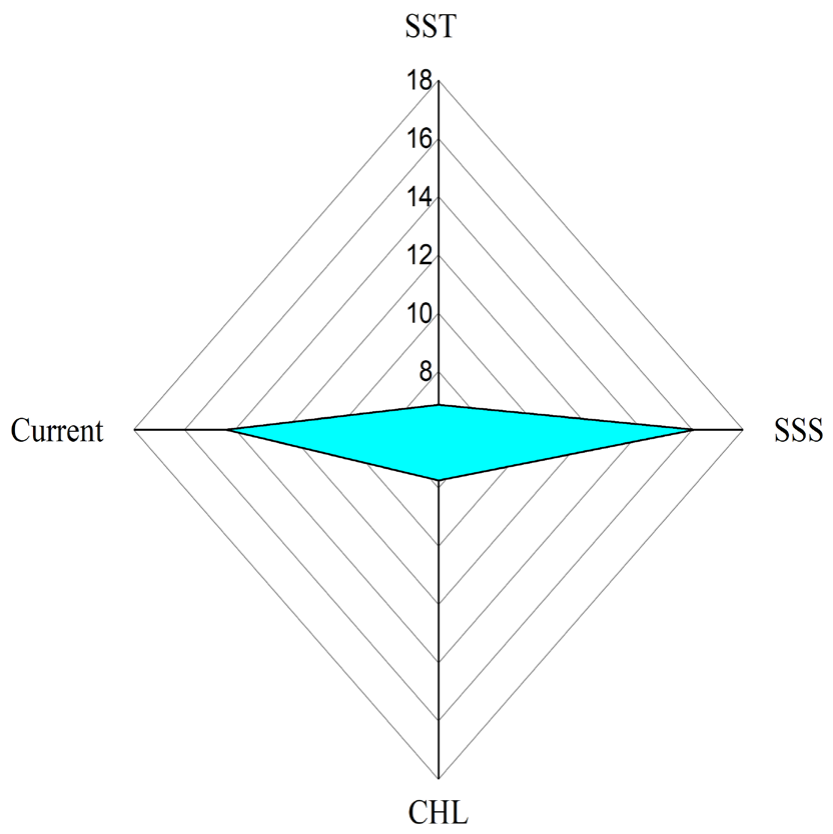
Percentage contribution of Sea Surface Temperature (SST), Chlorophyll-a Concentration (CHL-a), Sea Surface Salinity (SSS) and Current(s) relationship to mackerel catch.

The results of data processing from the catchment points, linked to environmental conditions (oceanographic parameters), reveal the relationship between the X and Y variables as illustrated in Fig. 4.

The results of the data visualisation indicated that salinity and currents were the parameters most significantly influencing mackerel catches contributing 16% and 14%, respectively (Fig. 4). Conversely, the parameters of sea surface temperature (SST) and chlorophyll-a (chl-a) exhibited lower contribution values. This aligns with the findings of Nugraha *et al*. (2020), which demonstrated that while SST is a critical factor in determining the presence of fish, it does not significantly influence the quantity or size of fish catches in the western part of the Banda Sea.

These findings are further contextualised by the peak fishing season (Fig. 2), which corresponds to the transitional period between the eastern and western monsoon seasons. During the period, increased rainfall leads to a significant decrease in salinity levels, stabilising surface salinity within the range of 30 psu–34 psu. This stability supports the reproductive activities of mackerel. Furthermore, transitional months such as November often experience shifts in currents and winds, potentially triggering upwelling in certain regions. This upwelling brings nutrient-rich, high-salinity water from deeper layers to the surface, enhancing primary productivity. The resulting abundance of nutrients serves as a critical food source for fish and mackerel larvae (*Rastrelliger* spp.).

### Prediction of Potential Mackerel Fishing Areas (*Rastrelliger* spp.) Based on ANN-Backpropagation

**FIGURE 5:**
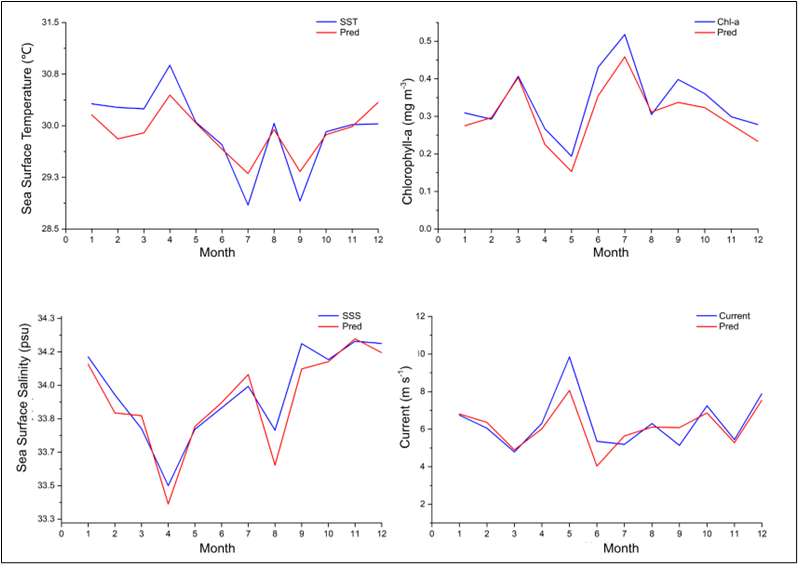
Comparative observation of model and actual data using MSE (Mean Squared Error) and RMSE (Root Mean Squared Error).

Fig. 5 shows a comparison between observed data (blue lines) and model-predicted data (red lines) for four key oceanographic parameters: sea surface temperature (SST), chlorophyll-a concentration, sea surface salinity (SSS), and ocean current speed over a one-year period (January–December).

In general, the four graphs illustrate that the model can represent the seasonal patterns of each parameter well. The fluctuation patterns of temperature, chlorophyll-a, salinity, and ocean currents shown by the predicted data follow the observed data trends, although there are slight differences in values in certain months, particularly during extreme conditions (maximum or minimum values).

This confirms that the prediction model has sufficient accuracy in depicting monthly oceanographic dynamics, making it suitable for understanding and projecting broader marine environmental conditions.

**FIGURE 6:**
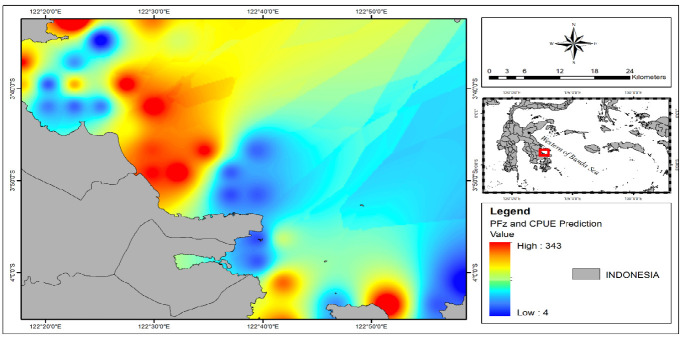
Prediction of potential mackerel fishing zones (Rastrelliger spp.) is based on the peak season (November 2020–2023) using the implementation of the Artificial Neural Network (ANN-Backpropogation) model.

In determining the potential fishing zones based on predictions, additional variables, including longitude, latitude and CPUE (Catch Per Unit Effort) predictions, were incorporated using the developed model. The results of the visualisation of the predicted potential mackerel fishing zones indicated that the parameters associated with the highest CPUE (343 kg trip^−1^) were observed at a sea surface temperature (SST) of 31.4°C, a chlorophyll-a concentration (CHL-a) ranging from 0.18 to 1.5 mg/m^3^, a current velocity of 3.29 cm/s and salinity (SSS) level of 34.2 psu (Fig. 6).

## DISCUSSION

The findings of this tudy demonstrate that the modelling of seasonal patterns and mackerel fishing grounds (*Rastrelliger* spp.) using the ANN algorithm in the western of Banda Sea provides valuable insights into the management of fishery resources. Data analysis from 2019 to 2023 revealed that November marks the peak of the fishing season, characterised by the highest CPUE and FSI values. These findings align with previous research, which established that seasonal variations in mackerel landings are positively correlated with chlorophyll-a concentrations (CHL-a) and negatively associated with SST.

Further analysis indicated that salinity (SSS) and ocean currents were the most significant oceanographic parameters affecting mackerel catches during this period, contributing 16% and 14%, respectively. This underscores the critical role of environmental conditions in determining the distribution and productivity of fish populations, including reproduction, which is often significantly influenced by salinity. Moreover, applying an ANN algorithm in this study proved to be highly effective, achieving a model accuracy exceeding 90%. The model successfully predicted fishing potential zones based on oceanographic parameters.

The integration of seasonal fishing index with ANN-based habitat suitability modelling offers a novel approach to identifying both temporal and spatial hotspots of mackerel abundance. By leveraging satellite-derived environmental data, this study provides a dynamic tool to guide fishers and policymakers in optimising fishing efforts while ensuring long-term sustainability. The combination of high-resolution CPUE data with oceanographic variability reinforces the model’s robustness in simulating real-world fishery dynamics. In addition, the results of this study provide a scientific basis for developing adaptive and ecosystem-based fisheries management strategies for Indian mackerel in the Banda Sea. To ensure the sustainable exploitation, several concrete policy recommendations are proposed implementation seasonal fishing regulations aligned with peak fishing months identified by the model to prevent overfishing during spawning seasons, establish dynamic catch limits that reflect seasonal variability in mackerel abundance as predicted by the model and designate marine protected areas in critical habitats such as spawning and nursery grounds to safeguard population regeneration. These measures align with the principles of sustainable fisheries and support the achievement of SDG 14 in Indonesia.

Furthermore, while the model was developed specifically for the western part of the Banda Sea, its high predictive accuracy and flexibility demonstrate its potential applicability to other fisheries in Indonesia and globally. The ANN approach can be adapted to model the habitat suitability of other species sensitive to oceanographic parameters, such as skipjack tuna, Sardinella lemuru and scad fish. Previous applications in different oceanographic contexts confirms the ANN’s versatility in supporting broader spatial fisheries planning. However, site-specific model recalibration and input parameter adjustment remain necessary to maintain accuracy and relevance across different ecological and geographic settings.

## CONCLUSION

This study developed a predictive model for the fishing season and potential fishing zones of Indian mackerel (*Rastrelliger* spp.) in the western of Banda Sea using an ANN approach. Based on analysis of catch data from 2019 to 2023, the peak fishing season for Indian mackerel occurs in November with the highest CPUE recorded at 220 kg/trip. This high catch rate signifies the aggregation of fish schooling during a biologically favourable period, likely linked to spawning activity and nutrient availability. As such, it represents a crucial temporal for optimising fishing efficiency while minimising pressure on the stock. This finding supports the development of seasonal management measures tailored to the biological rhythms of the species. Oceanographic parameters, such as salinity (34.2 psu) and current velocity (3.29 cm/s) were identified as the most influential factors, contributing 16% and 14%, respectively to the success of the catch.

The ANN model demonstrated a high level of accuracy (error rate of 1.12%) in predicting potential fishing zones based on oceanographic parameters. These zones were characterised by a sea surface temperature of 31.4°C, chlorophyll-a concentrations ranging from 0.18 to 1.5 mg/m^3^ and stable salinity conditions.

## Figures and Tables

**TABLE 1:** Oceanographic parameter data processed through satellite imagery.

No.	Parameter	Data source
1.	Sea Surface Temperature/SST (°C)	https://oceancolor.gsfc.nasa.gov/
2.	Chlorophyl-a/CHL-a (mg/m^3^)	https://oceancolor.gsfc.nasa.gov/
3.	Current (m/s)	https://data.marine.copernicus.eu/
4.	Sea Surface Salinity/SSS (psu)	https://data.marine.copernicus.eu/

**TABLE 2:** Intensity of the annual mackerel fishing season on 2019 to 2023 (five years) in western of Banda Sea.

No	Month	CPUE (kg/trip)	FSI (%)	± std. dev	Result
1	January	15.868	34.334	18.486	Regular season
2	February	40.353	93.609	61.259	Regular season
3	March	25.39	63.797	47.704	Regular season
4	April	41.148	107.242	66.15	Fishing season
5	May	27.536	59.427	24.521	Regular season
6	June	35.179	114.364	51.69	Fishing season
7	July	11.904	19.695	16.786	Lean season
8	August	58.849	108.364	54.022	Fishing season
9	September	125.25	178.093	110.056	Fishing season
10	October	65.76	82.51	63.014	Regular season
11	November	122.764	220.897	159.942	Peak season
12	December	71.883	117.667	93.508	Fishing season
